# Instrumentation of Stratospheric Balloon Straps with Optical Fibre for Temperature and Strain Monitoring

**DOI:** 10.3390/s20051433

**Published:** 2020-03-06

**Authors:** Yann Lecieux, Cyril Lupi, Dominique Leduc, Quentin Macé, Valentin Jeanneau, Pascale Guigue

**Affiliations:** 1 Laboratoire GeM (UMR CNRS 6183), Université de Nantes, CNRS, École Centrale de Nantes, 2, rue de la Houssinière, 44322 Nantes Cedex 3, France; cyril.lupi@univ-nantes.fr (C.L.); dominique.leduc@univ-nantes.fr (D.L.); quentin.mace@etu.univ-nantes.fr (Q.M.); valentin.jeanneau@etu.univ-nantes.fr (V.J.); 2 Centre National D’études Spatiales (CNES), 18 Avenue Edouard Belin, 31400 Toulouse, France; Pascale.Guigue@cnes.fr

**Keywords:** distributed fibre optic sensor, Brillouin scattering, thin structures, space structures, SHM

## Abstract

This article is devoted to the instrumentation, with optical fibres, of the straps holding the envelope of stratospheric balloons. This instrumentation is motivated in the first instance by the need to validate the numerical models used in the design of balloons. It must also be used to measure the temperature along the envelope in order to deduce the pressure field. It is shown at first that the optical fibres can be inserted inside a strap during its fabrication. Different kinds of insertion are considered, none of them perturb the industrial process. The instrumented straps were then submitted to thermal and mechanical tests and the distributed Brillouin frequency shifts were measured. We thus determined the type of insertion to be used according to the parameter (temperature or strain) to be measured and assessed the performance of the measurement chain.

## 1. Introduction

Stratospheric balloons have been used for a very long time to carry out scientific payloads: atmospheric monitoring measurements (e.g., temperature, pressure, CO_2_, presence of aerosols, wind, etc.) or astronomical measurements [1]. More recently, it has been proposed to use them to build a telecommunications network (Loon project). They consist of a gondola, a flight chain and an envelope, often a thin polyethylene film [2,3,4] reinforced with straps. There are different types of balloons, of different sizes and uses. This ranges from the 10 to 13 m diameter super pressure balloon that can carry about fifty kilograms at an altitude of 20 km, to the zero pressure stratospheric balloon whose envelope can have a perimeter of about two hundred meters and can carry up to 2 tons at an altitude of 40 km [5].

The design of stratospheric balloon envelopes is moving from an empirical approach to a more systematic approach that starts from scientific needs (payload, altitude, flight duration) and relies on mechanical models including laws of behavior of specific materials [6,7,8,9], to define the size of the envelope and the tests to be performed. During these tests, embedded sensors should measure the exact shape of the envelope. The comparison between the measured form and the numerical predictions should allow the models to be updated and thus should optimizes the reliability of the predictions of the numerical simulations. The measurement of the real shape is a key point of this rigorous design approach, hence the need for efficient instrumentation. In addition, to control or even pilot the balloon, it is crucial to know the pressure field inside. An indirect method to access this field would be to measure the temperature field, at least on the envelope.

Few experiments were conducted to measured these temperature and strain fields. Most of them used a camera to monitor the shape of the envelope [10,11]. This kind of measurement would hardly be used in flight given the size of the envelope of the balloon. To the contrary, Distributed Fibre Optic measurements seem particularly well suited for this type of monitoring. Indeed, the geometry of the optical fibres and their low weight make them well compatible with the thinness and size of the envelope. Distributed measurement techniques based on Brillouin [12,13], Rayleigh [14] or Raman [15,16] scattering make it possible to determine the strain or temperature throughout the perimeter of the envelope with a resolution of the order of one meter. Using reverse methods, the shape of the envelope can be determined from the strain measurements, or the pressure field from the temperature measurements. The first problem that arises is the insertion of the fibre into the envelope. As with any sensor, this is a critical problem: the fibre must not significantly modify the behavior of the structure, but at the same time a close link must be ensured so that the variation recorded by the fibre corresponds to that of the structure. In addition, the fibre insertion process must be compatible with the balloon manufacturing process. In this article we present a possible solution to this problem.

Section 2 is devoted to the description of the insertion of optical fibres into the straps that maintain the shape of the envelope during their manufacture. The straps thus instrumented are then subjected to thermal tests (Section 3) and mechanical tests (Section 4) to study the thermo-mechanical behavior of the instrumented balloon components. As a conclusion of these tests, recommendations are given on the method we consider optimal for inserting optical fibres into stratospheric balloon straps.

## 2. Insertion of Optical Fibres Inside Straps

**Figure 1:**
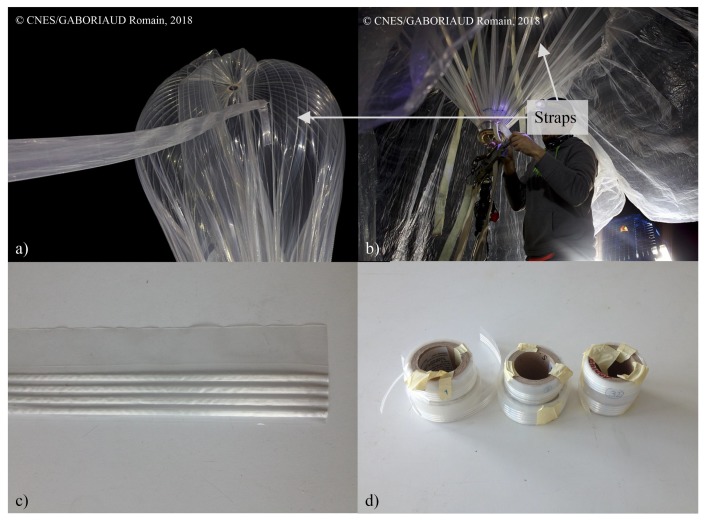
(**a**) Envelope of a stratospheric balloon. (**b**) Focus on strap assembly on the load ring. (**c**) Strap. (**d**) Different specimens of straps made on the balloon production line for the study.

The external envelope of a stratospheric balloon consists of a plastic film with a thickness of a few tens of micrometers (see Figure 1a). The plastic film is reinforced by straps consisting of an assembly of several strands of fibres which are assembled on the load ring (aluminum part) serving as a support for the balloon’s flight chain (see Figure 1b). It is thus the straps that support the load of the gondola. The weight of the load depends on the size of the balloon, from a few kilograms to several hundred of kilograms for balloons with diameters ranging from a few meters to several tens of meters in circumference.

**Figure 2:**
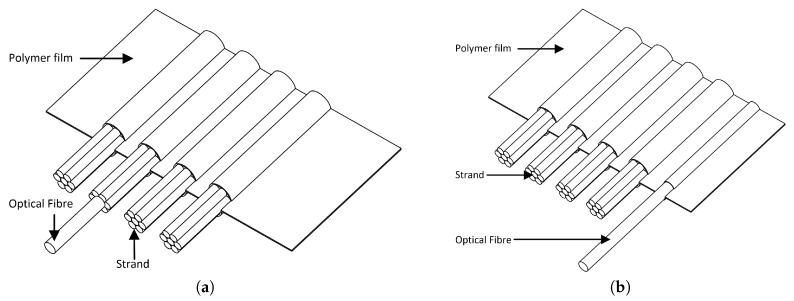
(**a**) Insertion of the optical sensor in a strand. (**b**) Insertion of the optical sensor between polymer films.

The considered straps are made of 4 strands of PET threads embedded between two 4.5 cm wide polymer tapes (see Figure 1c). The strands are spaced 3 mm apart. Three different kinds of optical fibres were tested and used in the framework of this study. Two were standard G652 fibres from Alcatel and Corning. The last one was a G657 fibre from Draka which can be submitted to bending with smaller radius. Different ways of inserting the fibre inside the straps were tested. The fibres were inserted as they were (bare fibres) or after being coated with a polyester resin. In both cases, they were inserted inside a strand or directly between two plastic tapes as shown on Figure 2. Finally, 7 specimens of instrumented straps were made (see Figure 1d) and named according to the following nomenclature: “Type of fibre-Location of insertion-Type of coating”:

- *Alcatel-Strand-Bare*: a G652 fibre from Alcatel inserted inside a strand;
- *Corning-Strand-Bare*: a G652 fibre from Corning inserted inside a strand;
- *Draka-Strand-Bare*: a G657 fibre from Draka inserted inside a strand;
- *Alcatel-Strand-Polyester*: a G652 fibre from Alcatel coated with polyester resin inserted inside a strand;
- *Alcatel-Film-Bare*: a G652 fibre from Alcatel inserted between two polymer films;
- *Alcatel-Film-Polyester*: a G652 fibre from Alcatel coated with polyester resin inserted between two polymer films;
- *Alcatel2-Strand-Bare*: two G652 fibre from Alcatel inserted in two different strands.

**Figure 3:**
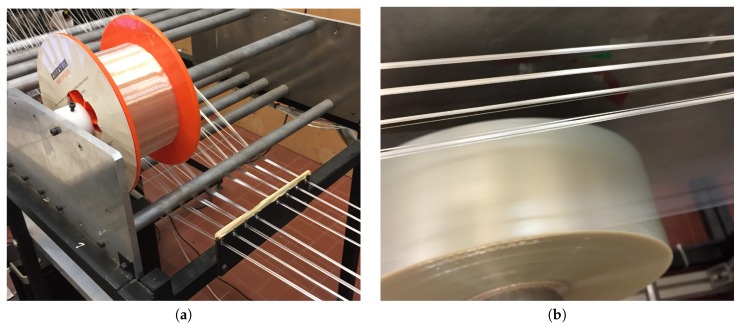
(**a**) Position of the optical fibre spoo. (**b**) View of the components of a strap: PET strand, optical fibre and polymer film.

The tests of insertion were performed on the straps production bench of CNIM Air Space (see Figure 3). The optical fibre spool has been positioned upstream of the assembly line on a support with a removable axle provided for this purpose. To insert the optical fibre into a strand, it is unwound and brought in about ten centimeters after the location where the two polymer films that make up the tape meet. The optical fibre is attached to the plastic tape with adhesive tape. Once the fibre is fixed and guided in the baffles, it is slightly tensioned again by winding the fibre support spool, and production is relaunched. During fabrication, plastic films are put under tension by a system of rollers and springs. It is therefore the strap that keeps the optical fibre in tension throughout the manufacturing process. It is interesting to note that no specific changes or adjustments need to be made on the production line following the assembly of the plastic films. The cutting and packaging of the straps is carried out in an unchanged manner compared to a standard strap manufacturing process, without any speed change. The strap manufacturing process including an uncoated polyester resin optical fibre can be described as quasi-industrial. In a few hours it was possible to produce several tens of meters of specimens of each type.

The only step in our experience that is a little bit artisanal was the coating of the fibres. It was done by unwounding the fibre by hand and immersing it in a bath of melted polyester. The coated fibre was then wound on a reel. This step was the most delicate because it had to be avoided that the fibre would stick back on itself. However, it was possible to make samples of 5 m with coated fibre. This process not allow to control the thickness of the polyester coating estimated at a few tens of microns.

The integrity of the inserted fibres was checked by simply connecting a light source at one end of the fibre and observing the light at the other end. None of them was broken except those in strap that has been bent with a radius of curvature of less than few millimetres during handling.

## 3. Thermal Tests

### 3.1. Thermal Calibration of Bare Fibres

**Figure 4:**
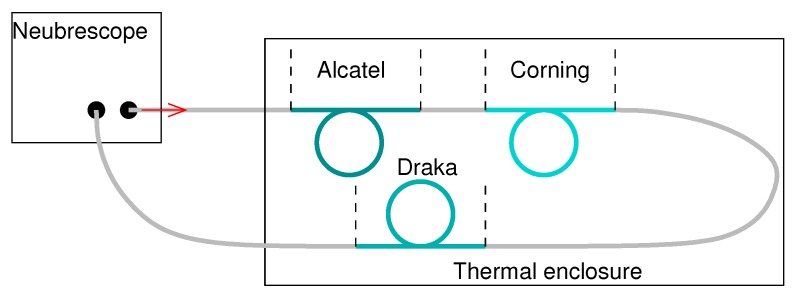
Assembly diagram of bare fibres.

**Figure 5:**
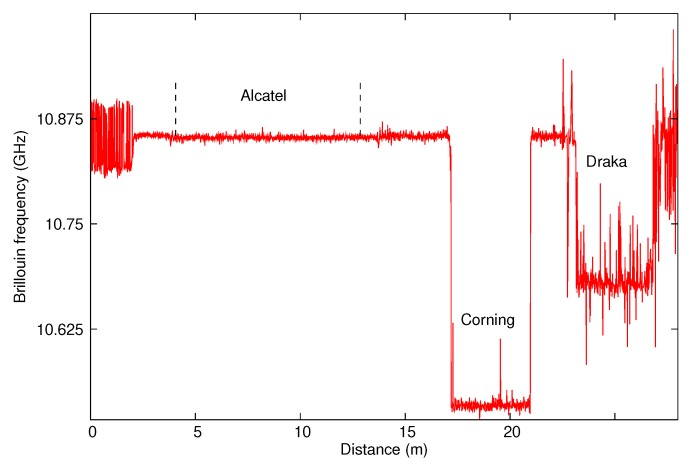
Measured Brillouin frequency along the bare fibres for a 20 °C temperature.

As first, the bare fibres sensitivities to temperature were calibrated. A sample of around 5 m was taken from each kind of fibre, wound on itself in such a way that it is free to deform with temperature. The three samples were then chained as shown on Figure 4 and placed inside a thermal enclosure Weiss WT 11 600. We then recorded the Brillouin frequency along the chain with a Neubrescope NBX-7020 in a Brillouin Time Domain Amplification (BOTDA) operating mode. An example of the recorded signal, obtained for a temperature of 20 °C, is presented on Figure 5.

The first sample is made of the same Alcatel G652 fibre as the link fibre so its response can not be distinguished from the background signal. Its position was determined using the hot spot method: we brought a heat source (extremity of a soldering iron) close to the splice and observed a localized peak in the Brillouin signal due to the increase in temperature, the localization of the peak gave the localization of the splice. It is located between 4.1 m and 13 m. In order to get its Brillouin frequency, we calculated the mean Brillouin frequency on the range [4.1 m;13 m] and the standard deviation was used to quantify the uncertainty. We then obtained a frequency of 10.852 ± 0.002 GHz. The second sample, made of Corning G652 fibre, is located between 23.4 m and 26.7 m. Its Brillouin frequency is 10.535 ± 0.006 GHz. The last sample, made of Draka G657 fibre, is located between 23.4 m and 26.7 m. The noise is higher for this measurement. It is assumed that the air convection current in the thermal enclosure caused the optical fibre to move during the measurement despite the fact that it was placed on a rigid support. The same phenomenon is not observed with others fibres placed at different location in the thermal enclosure. The Brillouin frequency is 10.68 ± 0.02 GHz. It should be noted that, for the three samples, the Brillouin frequency oscillates around a constant average value. This ensures that the temperature is constant along the samples and that the samples are free of stress.

**Figure 6:**
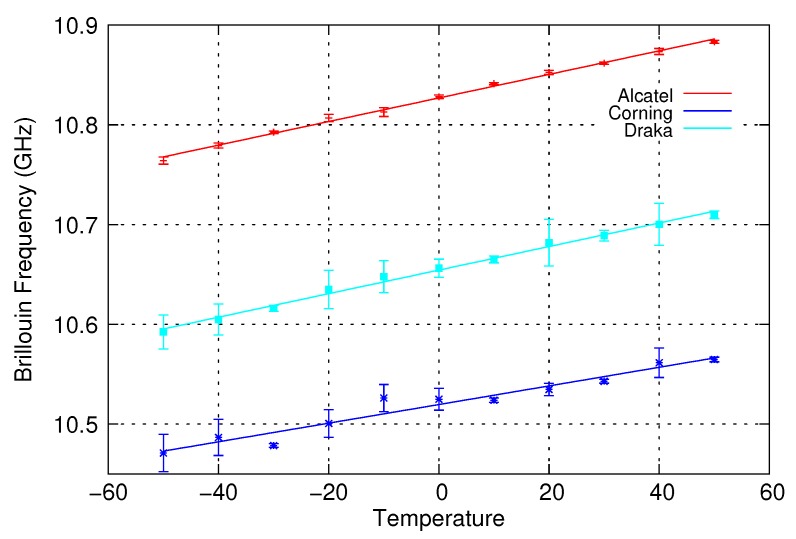
Variation of bare fibres Brillouin frequency with temperature.

The chain was characterized for temperatures ranging from –50 °C to +50 °C in steps of 10 °C with a minimum of 2 h left between two temperature levels to ensure temperature homogeneity in the chain. The resulting variations are shown in Figure 6. The points in this figure correspond to the average values measured with their associated standard deviation and the lines correspond to the linear fits made on these measurements. The Brillouin frequency varies linearly with the temperature in the considered range for all the samples. The coefficient of variation is 0.934 MHz/°C for the Corning fibre and 1.181 MHz/°C for Alcatel and Draka fibres.

### 3.2. Thermal Characterization of Instrumented Straps

**Figure 7:**
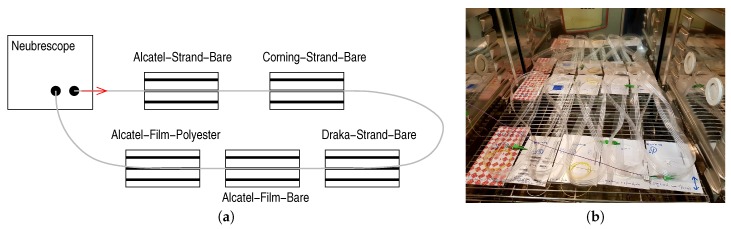
(**a**) Assembly diagram of straps. (**b**) Arrangement of the straps assembly in the thermal enclosure.

Samples of 2 m were taken from several instrumented straps. As for bare fibres, these samples were chained according to the diagram shown on Figure 7a. To minimize artefacts related to the shape of the straps, we placed the straps in the limited space of the enclosure, taking care to limit bendings and maximize flat surfaces as shown on Figure 7b.

**Figure 8:**
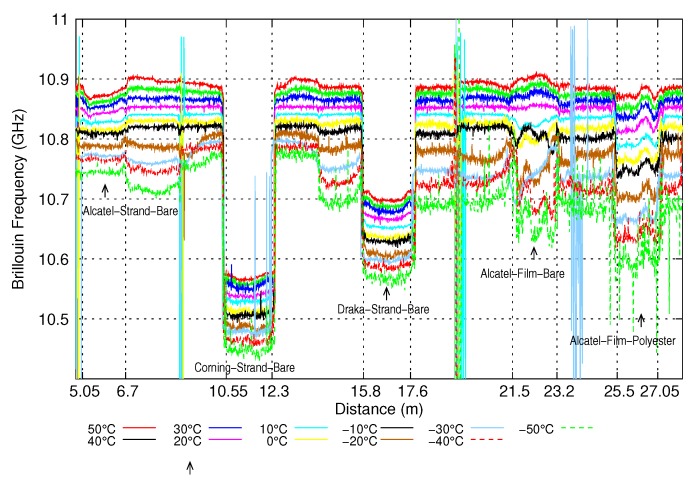
Variation of the Brillouin frequency of straps with temperature.

The straps were submitted to the same thermal tests as the bare fibres. Figure 8 shows the Brillouin frequency profiles measured for the different temperatures. Sample *Alcatel-Strand-Bare* is located between 5.05 m et 6.7 m, sample *Corning-Strand-Bare* between 10.55 m and 12.3 m, sample *Draka-Strand-Bare* between 15.8 m and 17.6 m, sample *Alcatel-Film-Bare* between 21.5 m and 23.2 m and sample *Alcatel-Film-Polyester* between 25.5 m et 27.05 m.

It can be seen that the Brillouin frequency is less uniform along each sample as it was along bare fibres. This is due to the stress exerted by the strap on the optical fibre during the strap’s production process. However, the shape of the Brillouin frequency profile remains almost the same when the temperature changes, that is, if we subtract the profile obtained at 0° to the profile at temperature *T*, we obtain a curve almost flat, varying around a constant value. This average value is taken as the Brillouin frequency shift and the standard deviation as the uncertainty on the Brillouin frequency shift.

**Figure 9:**
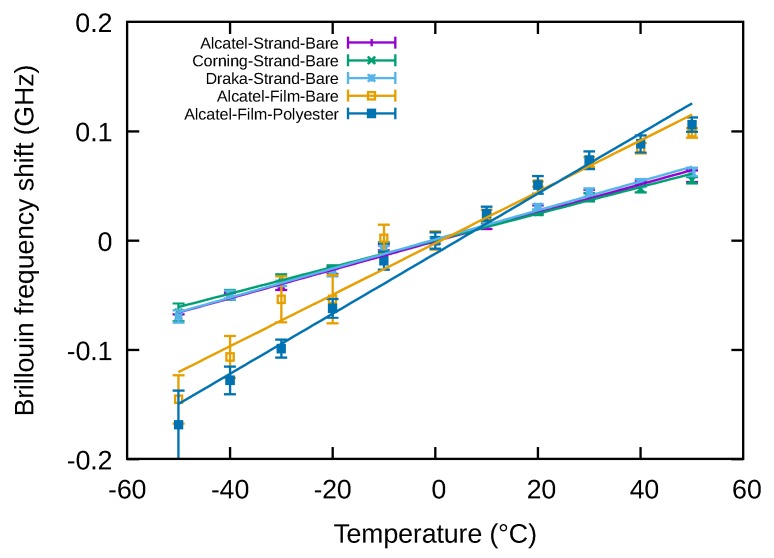
Variation of Brillouin frequency shift with temperature.

The Brillouin frequency shifts of the different samples are shown on Figure 9. The points correspond to the measurements and the line to the linear fits. The coefficients of variation of Brillouin frequency shifts with temperature of the different samples are listed in Table 1 and compared to those of bare fibres.

**Figure 10:**
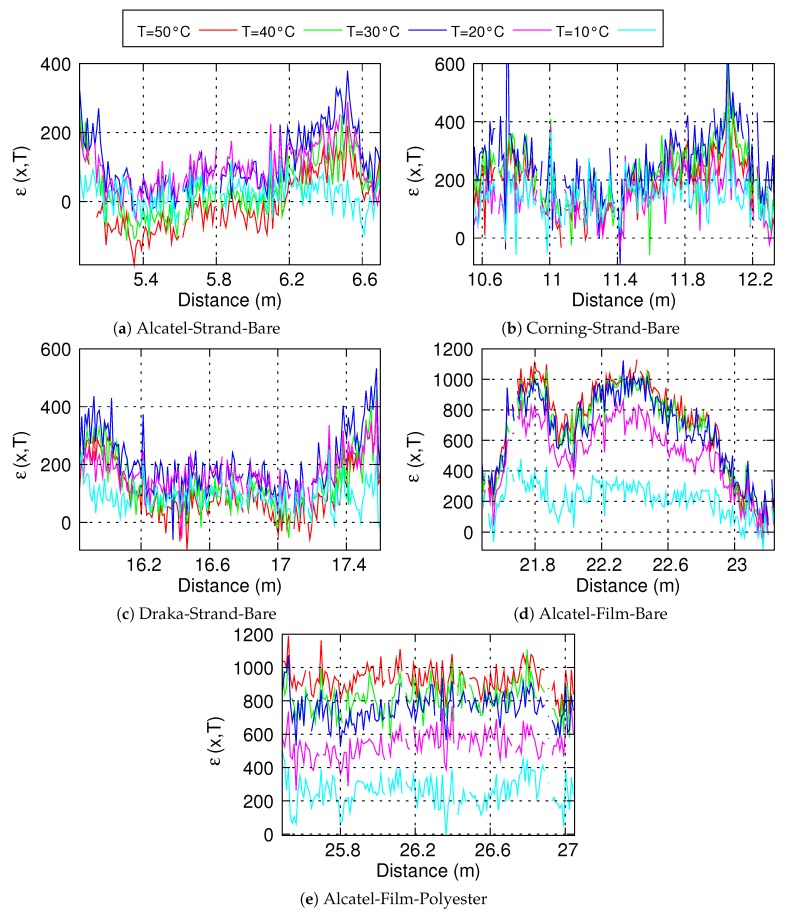
Strap induced strain for the different samples and different temperatures (**a**–**e**).

When the fibre is inserted inside a strand, its response with temperature is close to that of the bare fibre, whereas it is significantly different when the fibre, either coated with polyester or not, is inserted between two polymer films. This means that, in the strand, the fibre is free to slip. Between the films, it seems to be pulled by the strap which thermal expansion coefficient is higher. In order to qualify the adhesion between the fibre and the strap, we computed the strain, ε(x,T), induced by the strap:

(1) $$\varepsilon \left(x,T\right)=\left[\nu_{B}\left(x,T\right)-\nu_{B}\left(x,0\right)-\alpha_{\mathrm{bare}\;\mathrm{fibre}}^{B}T\right]/C_{\varepsilon }$$

where *x* is the distance along the fibre, *T* the temperature, $\nu_{B}\left(x,T\right)$ the measured Brillouin frequency and $C_{\varepsilon }$ the coefficient of variation of the Brillouin frequency with strain. The results are shown on Figure 10.

The strap induced strain for fibres inserted inside a strand (Figure 10a–c) does not depend on the temperature. This confirm the assumption of the slipping of the fibre inside the strap. For the bare fibre inserted between two polymer films (Figure 10d), the strap induced strain increases with temperature from 0 °C to 20 °C and then does not evolve much. We can assume that the fibre is pulled by friction, but that there is no real bonding. In the last case (Figure 10e), we observe an almost flat strap induced strain, which increases linearly with the temperature. This indicates that the fibre is really bonded to the strap.

Since the fibre is not bonded to the polymer film for cases presented in Figure 10a–d, it could have been expected to have a strain-free optical fibre and thus measure only thermal expansion effects on the fibre. This is not the case, but it is not the main problem. The main problem is that the contact and friction resulting from the polyester fibres or the polymer film on the optical fibre induces a strain gradient that varies with temperature. This phenomenon is likely to induce measurements interpretation errors. Indeed, the level of strain measured will depend on the change in temperature, the tension in the strap and the friction. However, in the case of an *in-situ* measurement, it will not be possible to dissociate the three effects. It can also be seen that the different fibres tested and thus the different constitution of the outer coating do not make any differences. It cannot be said that one coating is more effective than another in reducing friction. Only the assembly of the optical fibre glued on the polymer film thanks to a polyester coating makes it possible to obtain a really usable measurement (see Figure 10e). In this case, the stress shift at any point of the optical fibre depends linearly and solely on the temperature increase due to the thermal expansion of the polymer film to which it is bonded.

## 4. Mechanical Tests

### 4.1. Mechanical Calibration of Bare Fibres

**Figure 11:**
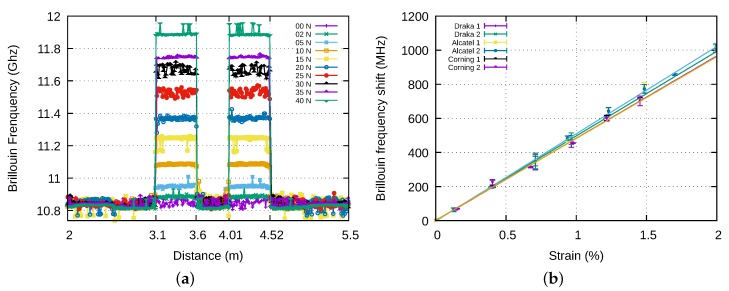
(**a**) Brillouin frequency profile for Alcatel fibres. (**b**) Variation of the Brillouin frequency with strain.

The sensitivity of bare fibres to strain was first calibrated. The mechanical tests were performed with a Zwick tensile testing machine of 30 kN capacity. Two samples were taken from each kind of fibre. Each sample was uncoated over 20 mm in two areas separated by 50 cm. The uncoated areas were then bonded with an epoxy resin to metal parts serving as clamping part in the jaws of the tensile testing machine. This way ensures a perfect bonding between the fibre and the jaws. The elongation of the fibre is therefore equal to the displacement of the moving crosshead. A couple of the same kind of fibres was stuck on the clamping parts for each test. The tensile testing machine was driven by force. The force was increased by steps of 25 N. At each step, a measurement of the Brillouin frequency profile was made with the Neubrescope. The two fibre samples were linked by one end. They therefore appear successively on the measured profile, as shown on Figure 11a.

In order to analyze the measurements, the same method as for thermal tests was used: the Brillouin frequency of a sample is given by the mean value of the measured Brillouin frequency along the sample and the corresponding uncertainty is the standard deviation over the same range. Results are shown on Figure 11b. As expected, the Brillouin frequency varies linearly with the strain for each fibre. The variation coefficients are: $C_{\varepsilon }=513\pm 6$ MHz/% for the Alcatel fibre, $C_{\varepsilon }=480\pm 12$ MHz/% for the Corning fibre and $C_{\varepsilon }=490\pm 5$ MHz/% for the Draka fibre. These values are close to those of the literature [17].

### 4.2. Mechanical Tests of Instrumented Straps

**Figure 12:**
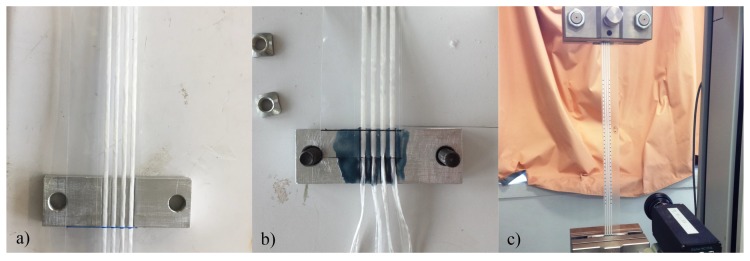
(**a**) Assembly of clamping parts and straps before bonding according to method 1. (**b**) Assembly of clamping part and straps according to method 2: the film is bonded over half the width of the clamping parts and the strands and the fibre are bonded over the other half. (**c**) Assembly of a test specimen in the tensile machine.

The first issue is the way to fix the strap to the moving crosshead of the tensile testing machine. Different ways of bonding the straps to clamping parts tightened in the tensile testing machine jaws were tested. In the first one, the polymer film only is bonded on the metallic clamped part. The strands of PET fibres and the optical fibre are therefore simply held between the two films by the sizing made during manufacture. This is the closest thing to the actual mounting on stratospheric balloons. In the second method, the plastic film is cut at its ends so that the strands and the optical fibre protrude. The film is bonded over half the width of the jaws and the strands and the fibre are bonded over the other half. The displacement imposed by the tensile testing machine is therefore the same for each component of the strap (film, strand and optical fibre). This way of assembling the different components of the straps is equivalent to adding epoxy glue on the loading ring of the balloon visible in the Figure 1b on which the straps are assembled. Finally, when the optical fibre is glued to the polymer film thanks to the polyester coating, only the polymer film and the strands are glued to the clamping parts. The metallic clamped part, the two mounting systems and the assembly of a tested specimen in the tensile testing machine jaws are shown on Figure 12.

Two to three samples of 50 cm long were tested from the following specimens: *Alcatel-Strand-Bare, Alcatel2-Strand-Bare, Draka-Strand-Bare* and *Alcatel-Strand-Polyester*. As for bare fibres, the force imposed by the tensile testing machine was increased by steps of 25 N and the Brillouin frequency profile was measured at each step. The mean value of the Brillouin frequency shift over the length of the strap and the uncertainty on this value were computed as for bare fibres.

**Figure 13:**
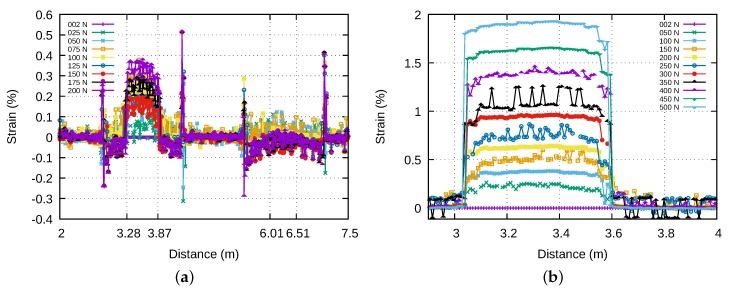
Strain measured by optical fibres inserted in straps. In (**a**) only the polymer film is bonded to clamping parts. In (**b**) the optical fibre is bonded with the polyester coating to polymer film while the polymer film and the strands are bonded to clamping parts.

**Figure 14:**
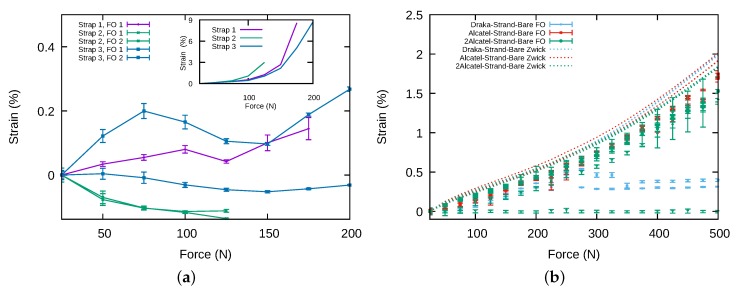
(**a**) Strain measured by the tensile testing machine and the optical fibres when the polymer film only is bonded to clamping parts. (**b**) Strain measured by the optical fibre when the polymer film, the optical fibres and the strands are bonded to clamping parts (FO denotes strain measured by optical fibres and Zwick strain measured by testing machine).

The *Alcatel2-Strand-Bare* specimens contain 2 optical fibres. These fibres were connected by one end, in order to get a single profile with the Neubrescope. This can be observed on Figure 13a where the signal between 3.28 m and 3.87 m corresponds to the light travelling inside the first fibre of the strap in the forward direction, and the signal between 6.01 m and 6.51 m to the light travelling in the second fibre of the strap in the backward direction. The *Alcatel-Strand-Polyester* specimen shown in Figure 13b contains a single optical fibre located between 3.1 and 3.6 m. Figure 14a shows the variation with the imposed force of the strain measured by optical fibres for three samples of *Alcatel2-Strand-Bare* specimen using the bonding to polymer film only method. The insert shows the strain measured by the tensile testing machine which serves as reference. It can be seen that the strains experienced by the fibres are not correlated to that of the strap. Moreover, the strains measured by two fibres inside the same strap are not even the same. This appears also on Figure 13a where the first fibre is clearly strained by the strap whereas the second fibre is not. By contrast, when the optical fibre is bonded to the polymer film, as is the case for the specimen shown in Figure 13b, the strain level is practically constant from one end of the optical fibre to the other, as is assumed to be the case in pure tensile stress.

The first observation is that when the external plastic film only is bonded at its two ends, the optical fibre is strained by the strap in an erratic way. The strain experienced by the fibre is far smaller than the strain of the strap. This assembly method does not give reliable information on the strain of the balloon envelope, i.e., the plastic film. However, there is also no indication that the sensor undergoes the same elongation as the fibre strands. Moreover, if we look back to the Brillouin frequency, the shifts are of the order of 10 to 100 MHz. These shifts correspond to a variation of temperature of the order of 10 to 100 °C. This mean that the strain of the fibre may conceal a temperature variation. Therefore, as explained in the Section 3, this type of assembly is also not suitable for temperature measurement using a single Rayleigh or Brillouin scattering measurement. In order to perform a temperature profile measurement with this kind of bonding, it is necessary to use a method insensitive to strain such as Raman scattering or to combine two methods, for example Rayleigh and Brillouin scattering, to de-correlate strain and temperature.

Since the first method of bonding the strap to the clamping parts failed in giving accurate strain measurements, we have chosen to bond the polymer film, the fibre strands and the optical fibre to the clamping parts to perform the mechanical tests presented afterwards. Figure 14b summarises the results obtained with bare fibres inside strap. This time the strains measured by the fibres (FO in legend) and by the machine (Zwick in legend) are plotted together since they have the same scale. For Alcatel fibres, but one, the measured strain is close to the strain measured by the tensile testing machine. Both present the same increasing behaviour with the force but the strain measured by the fibres is always 10% to 20% smaller. For Draka fibres, the measured strain is correct up to 250 N (or 0.5% in strain) and then becomes erroneous. This is due to a debonding between the coating and the silica part of the fibre. The sensing part of the optical fibre can then slip inside the strap and its strain becomes more or less independent from the strain of the strap.

**Figure 15:**
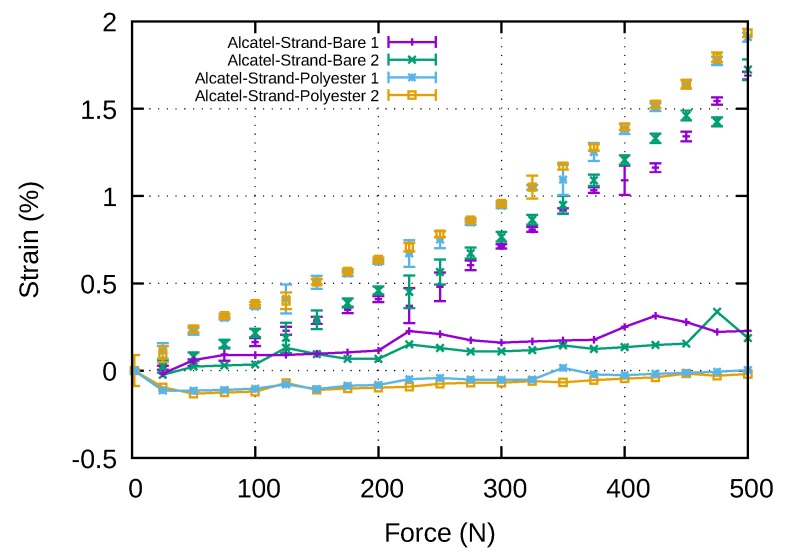
Strains measured by bare and coated fibre inside strap (points) and difference with the strain measured by the tensile testing machine (lines-points).

Finally, the Figure 15 shows the comparison of the strain measurements performed with a bare Alcatel fibre and an Alcatel fibre coated with polyester. The points corresponds to raw fibres strain measurements and the lines with points to the difference between fibres measurements and tensile testing machine measurements. As expected, the measurements made with polyester coated fibres are closer to the machine measurements especially for higher strains. Optical fibres coated with polyester to ensure adhesion with the polymer film should therefore be preferred for instrumentation where high accuracy is required. However, it is important to note that the thickness and the type of the bonding agent can significantly affect the strain transfer mechanism between the host material and the optical fibre (e.g., optical fibre to balloon envelope or optical fibre to clamping parts); thus, part of the reported deviations may also be attributed at this effect. This phenomenon is described in the reference [18]. Thus, as part of the industrialisation of the strap manufacturing process, a specific study should be carried out on the choice of these two parameters.

## 5. Conclusions

In this paper, we presented a study on optical fibre instrumentation of stratospheric balloon straps. This work is a necessary first step towards balloon envelope monitoring. This is a subject that has been rarely studied to date, but which would nevertheless allow to optimize the geometry of the envelope or to improve the control of balloons in flight. For these two applications, optical fibres seem to be a particularly suitable solution. Thus, we have shown that it is possible to insert one or even two optical fibres, bare or coated with polyester, into the straps during their manufacture, without slowing down or modifying the production line. The only step that remains to be automated from an industrial perspective is the coating of the optical fibre with polyester.

Thermal tests have shown that, in strain-free straps, the uncoated optical fibres slide while the polyester-coated fibres follow the thermal dilatation of the strap. Mechanical tests were then carried out to determine the behaviour of the optical fibres inserted in the straps attached at both ends. Two types of bonding were considered. In the first one, only the polymer film of the strap is bonded to the clamping parts tightened in the jaws of the tensile testing machine. This replicates the way the straps are attached in a real balloon. Tensile tests show that in this case the bare fibres slide inside the straps. The measurements show erratic but possibly large strains that cannot be correlated with strap tension. Therefore, these fibres cannot be used to measure the level of tension in the strap. If they are used to make a temperature profile measurement, a method that is insensitive to strain must be used, or a means must be available to estimate the residual strains so as to eliminate their influence on the Brillouin frequency shift. A temperature measurement by Raman effect could also be considered if temperature is the only quantity of interest.

In the second type of bonding tested, the fibres and polymer film were bonded to the clamping parts. With this configuration, the strain measurement given by the optical fibres follows the strain of the strap well. The relative error remains between 10% and 20% for bare fibres but falls below 5% for polyester-coated fibres as soon as the strain exceeds 10,000 με. This configuration can then be used to perform accurate strain measurements of stratospheric balloon straps. Moreover, an additional advantage of opting for a sensor with a coating adhesive to the film is that the envelope strain can be measured locally at any point on the balloon. By contrast, two-point bonding will only allow the average film tension to be measured, which somewhat limits the interest of this measurement. The technical solution of bonding the optical fibre to the balloon envelope film, even if it is complicated to achieve, is therefore to be preferred for measurements of strains by Brillouin or Rayleigh effect, or in the context of combined measurements in order to dissociate strain and temperature. This study is directly transposable to the study of a real balloon, which will be the next step of this work. The main difference will be the spatial resolution of the measurement. In this study strain measurements with a measurement uncertainty of 10 μm/m (data from NEUBREX company) were performed on 50 cm straps with a spatial resolution of 2 cm. For systems longer than 100 m, this spatial resolution will increase to 50 cm.

In a later stage, after mock-up testing, a Brillouin Rayleigh interrogator could be placed in the gondola of a balloon to evaluate the interest of using optical sensors for flight control. Carrying equipment weighting a few dozen kilos, i.e., the weight of an interrogator, batteries and a heating system does not present any technical difficulties. Balloons can carry several hundred kilograms of equipment (3.6 tons for the largest model used by NASA according to [5]). They are currently equipped with a heating system allowing the operation of electronic equipment at high altitude. Finally, even if optical interrogators are expensive, their cost remains low compared to the cost of observation instruments placed in the stratospheric balloon gondolas. In addition, techniques exist for recovering scientific equipment from balloons at the end of a flight and reusing it for next flights (see [5]). If the results of this phase are convincing, the miniaturization of the measuring devices could be envisaged. The experience obtained from distributed measurements could also be used to define the location and number of sensors needed for punctual measurements, e.g., with fibre Bragg grating sensors with light, much cheaper and energy-efficient interrogators. Indeed, it is possible to have custom-made optical fibres with punctual sensors at defined locations. However, at this stage of technological development, these sensors are too expensive to use. It is preferable to use a Brillouin interrogator and standard optical fibres to test the insertion of the fibres into the strands, the making of balloons, and to acquire measurements over the entire surface of a balloon.

## Figures and Tables

**Table 1:** Coefficients of variation of Brillouin frequency shift with temperature.

	Sample				
	Alcatel-Strand-Bare	Corning-Strand-Bare	Draka-Strand-Bare	Alcatel-Film-Bare	Alcatel-Film-Polyester
$\alpha_{\mathrm{strap}}^{B}$ (MHz/°C)	1.30	1.22	1.33	2.3	2.7
$\frac{\alpha_{\mathrm{strap}}^{B}-\alpha_{\mathrm{fibre}}^{B}}{\alpha_{\mathrm{fibre}}^{B}}$	0.1	0.3	0.13	0.95	1.3
